# Immunoenhancing Effects of *Cyclina sinensis* Pentadecapeptide through Modulation of Signaling Pathways in Mice with Cyclophosphamide-Induced Immunosuppression

**DOI:** 10.3390/md20090560

**Published:** 2022-08-31

**Authors:** Rui Zhao, Xiao-Xia Jiang, Qiao-Ling Zhao, Han-Wei Ye, Yi Lin, Ju Huang, Yun-Ping Tang

**Affiliations:** 1 Zhejiang Provincial Engineering Technology Research Center of Marine Biomedical Products, School of Food and Pharmacy, Zhejiang Ocean University, Zhoushan 316022, China; 2 Zhoushan Institute for Food and Drug Control, Zhoushan 316000, China; 3 Key Laboratory of Health Risk Factors for Seafood of Zhejiang Province, Zhejiang Ocean University, Zhoushan 316022, China

**Keywords:** *Cyclina sinensis*, pentadecapeptide, immunomodulatory, cyclophosphamide, mechanism

## Abstract

Our study aimed to investigate the immune-enhancing mechanism of the pentadecapeptide (RVAPEEHPVEGRYLV) from *Cyclina sinensis* (SCSP) in a cyclophosphamide (CTX)-induced murine model of immunosuppression. Our results showed that SCSP treatment significantly increased mouse body weight, immune organ indices, and the production of serum IL-6, IL-1β, and tumor necrosis factor (TNF)-α in CTX-treated mice. In addition, SCSP treatment enhanced the proliferation of splenic lymphocytes and peritoneal macrophages, as well as phagocytosis of the latter in a dose-dependent manner. Moreover, SCSP elevated the phosphorylation levels of p38, ERK, JNK, PI3K and Akt, and up-regulated IKKα, IKKβ, p50 NF-κB and p65 NF-κB protein levels, while down-regulating IκBα protein levels. Our results indicate that SCSP has immune-enhancing activities, and that it can activate the MAPK/NF-κB and PI3K/Akt pathways to enhance immunity in CTX-induced immunosuppressed mice.

## 1. Introduction

The immune system, composed of a complete set of immune organs, cells, and active substances, constantly monitors the body for foreign entities and maintains the continuous and healthy operation of the entire body [1]. However, factors such as obesity [2], stress [3], mood [4], and lifestyle [5] have been shown to affect its normal functioning. Immunotherapy can artificially enhance or suppress the body’s immunological responses in cases of low or hyperactive conditions in order to return the immune system to its physiologic status [6]. Compared with the instability and adverse effects of chemically synthesized immunomodulators, natural products with immunomodulatory activity offer a way of effectively avoiding these risk factors [7,8]. Therefore, it is necessary to identify safe and effective natural immune modulators. 

In the field of natural active product development, the marine environment offers a treasure trove of natural compounds with distinctive biological characteristics, due to its high biodiversity and complex ecological relationships [9]. Marine peptides have attracted much attention due to their unique biological properties, such as antihypertensive [10], antioxidant [11], antitumor [12], and antidiabetic activities [13]. Immunomodulatory peptides identified from different marine sources have shown significant immune-enhancing activities [14,15,16]. For example, Li et al. [17] purified two peptides (DNSIAMESMK and LLQLGSG) from oyster hydrolysate and showed that these two peptides markedly promoted the proliferation of murine lymphocytes and the phagocytic ability of macrophages. Cai et al. [18] isolated two peptides (HIAEEADRK and AEQAESDKK) from trypsin hydrolysates of tuna and showed that these two peptides could bind to the active sites of TLR2 and TLR4 and stimulate macrophage activation. Xu et al. [19] purified a peptide (YVMRF) with immunoregulatory activity from *Stolephorus chinensis* and confirmed that YVMRF could stimulate RAW 264.7 differentiation and increase the concentrations of nitric oxide (NO), TNF-α, IL-6, and IL-1β. In previous studies, we purified an immunomodulatory peptide (RVAPEEHPVEGRYLV) from *Cyclina sinensis* (SCSP) and demonstrated that SCSP showed significant immune-enhancing activities in mice with CTX-induced immunosuppression [20]. However, the mechanisms underlying the immunomodulatory effects of SCSP have not been elucidated. 

Several signaling pathways have been shown to play crucial roles in immune activation, including MAPK, PI3K/Akt, and downstream NF-κB pathways [21,22,23]. Yu et al. [24] demonstrated that sulfate-modified *Cyclocarya paliurus* polysaccharide could enhance the secretion of TNF-α, IL-10 and NO in immunosuppressed mice by modulating the MyD88-dependent MAPK/NF-κB/PI3K-Akt signaling pathway. He et al. [25] verified that low-molecular-weight peptides from *Mytilus coruscus* exerted immunomodulatory effects on macrophages by regulating the NF-κB/MAPK pathway. Yao et al. [26] reported that European eel (*Anguilla anguilla*)-derived peptides promoted the production of NO, inducible nitric oxide synthase (iNOS) and cytokines by modulating the NF-κB and MAPK pathways in macrophages. Moreover, the hexapeptide RNPFLP isolated from *Lepidium meyenii* protein hydrolysate activated RAW 264.7 cells via TLR2 and TLR4 receptor-mediated activation of the NF-κB and MAPK pathway [27]. In this study, we focused on the MAPK/NF-κB and PI3K/Akt pathways to investigate the potential mechanisms underlying the immune-enhancing effects of SCSP in mice with CTX-induced immunosuppression. Our results provide an explanation for the effects of SCSP and support its use as a novel immunomodulator candidate or immune adjuvant.

## 2. Results

### 2.1. Effect of SCSP on Immune Organ Indices

**Figure 1:**
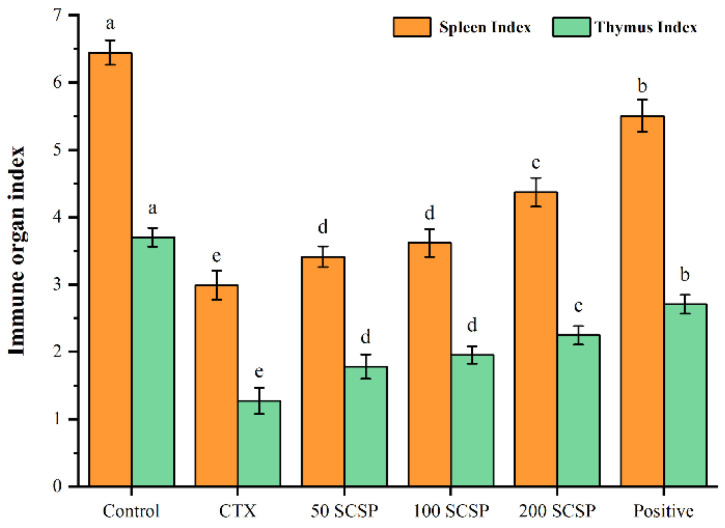
Effect of SCSP on immune organ indices in CTX-induced mice (*n* = 10). Different letters over bars indicate statistical significance between two groups (*p* < 0.05), the same as below.

Body weight and organ indices are often utilized as the primary metrics to examine the physiological conditions of experimental animals and the therapeutic impact of medications in the early stages of research [28]. Our previous studies showed that the final murine body weight in the SCSP-treated groups was significantly higher than in the CTX group, suggesting that SCSP could improve CTX-induced murine body weight loss [29]. Moreover, SCSP treatment effectively increased the murine immune organ indices, showing that SCSP could alleviate immune organ damage by CTX (Figure 1).

### 2.2. Effect of SCSP on Cytokine Production

**Figure 2:**
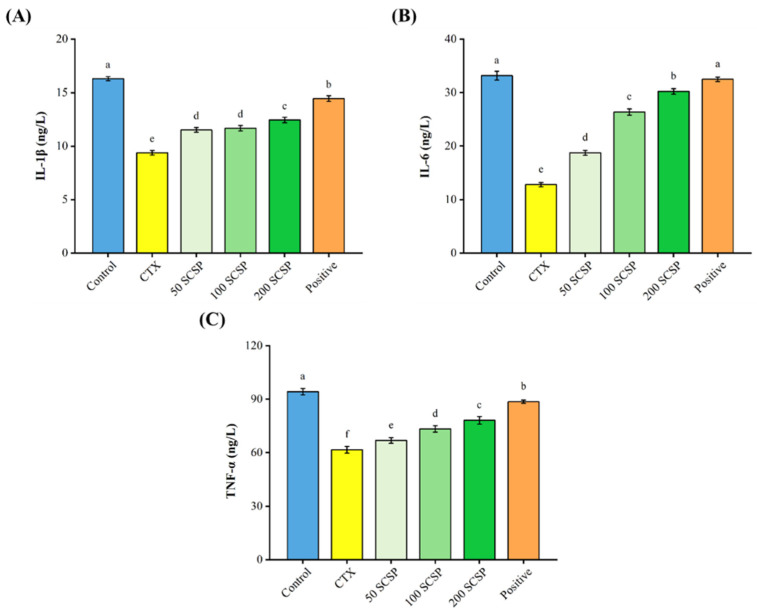
Effect of SCSP on serum levels of IL-1β (**A**), IL-6 (**B**), and TNF-α (**C**) in immunosuppressed mice (*n* = 10).

As shown in Figure 2, CTX significantly inhibited the secretion of IL-1β (9.38 ± 0.21 ng/L vs. 16.30 ± 0.19 ng/L), IL-6 (12.79 ± 0.39 ng/L vs. 33.16 ± 0.82 ng/L), and TNF-α (61.61 ± 1.88 ng/L vs. 94.27 ± 1.75 ng/L) when compared with the control group. Remarkably, higher concentrations of the three cytokines were observed (12.44 ± 0.26 ng/L, IL-1β; 30.22 ± 0.50 ng/L, IL-6; and 78.20 ± 2.12 ng/L, TNF-α) after treatment with 200 mg/kg of SCSP, although it was still lower than in the positive control group. The above results suggest that SCSP has an antagonistic effect on CTX-induced suppression of cytokine secretion.

### 2.3. Effects of SCSP on Cellular Immunity

**Figure 3:**
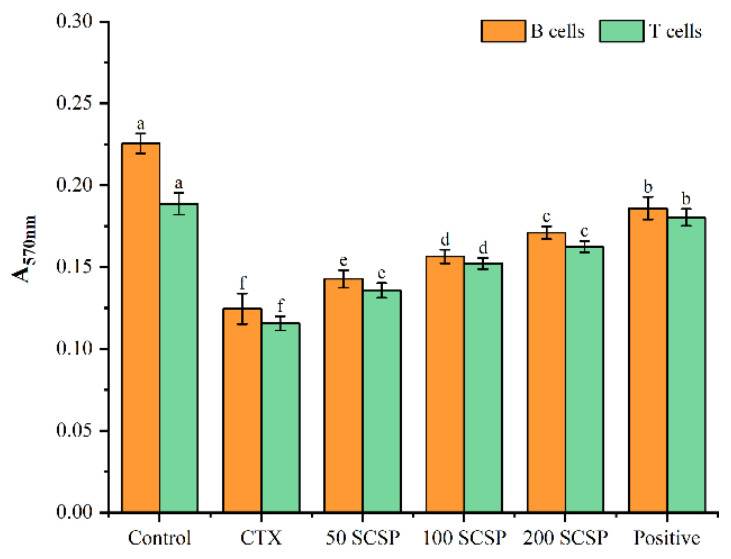
Effect of SCSP on the proliferative capacity of mouse spleen lymphocytes in vitro (*n* = 10).

An experiment with splenic lymphocytes was carried out to determine the impact of SCSP on T and B cellular immune responses. As shown in Figure 3, CTX treatment significantly inhibited the proliferative activity of these two immune cell populations when compared with the control group (0.125 ± 0.009 vs. 0.226 ± 0.006, B cells; 0.116 ± 0.004 vs. 0.189 ± 0.007, T cells; *p* < 0.05). However, when animals were treated with different concentrations of SCSP, the proliferation of B and T cells improved substantially when compared with the CTX group (*p* < 0.05), indicating that SCSP enhanced cellular immune responses, increasing spleen lymphocyte proliferation. Otherwise, it was still lower than in the positive control group.

**Figure 4:**
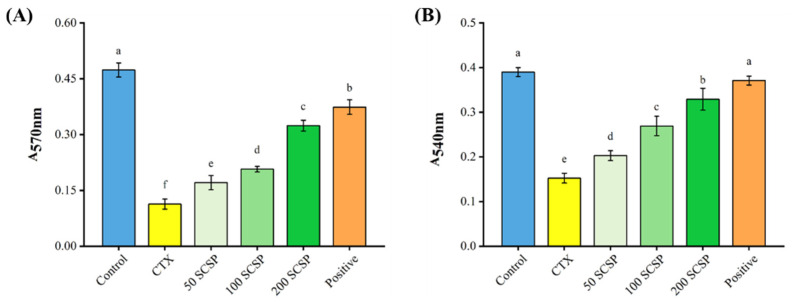
Effect of SCSP on the proliferative (**A**) and phagocytic (**B**) capacity of peritoneal macrophages in mice (*n* = 10).

Proliferation (Figure 4A) and phagocytosis (Figure 4B) of mouse peritoneal macrophages were also analyzed to examine the regulatory effects of SCSP on immune cells. CTX significantly inhibited the proliferation and phagocytic activity of macrophages when compared with the control group. However, with increasing doses of SCSP, the proliferative rate of macrophages gradually increased, reaching its highest at 200 mg/kg of SCSP (0.324 ± 0.014), although it was still lower than in the positive control group (0.374 ± 0.019). On the other hand, SCSP considerably restored the phagocytic ability of macrophages when compared with the CTX-treated group, and this effect reached its highest at 200 mg/kg of SCSP. However, it was still lower than in the positive control group. These findings demonstrate that SCSP can enhance lymphocyte and macrophage activity to overcome CTX-induced immunosuppression.

### 2.4. Western Blot Analysis

**Figure 5:**
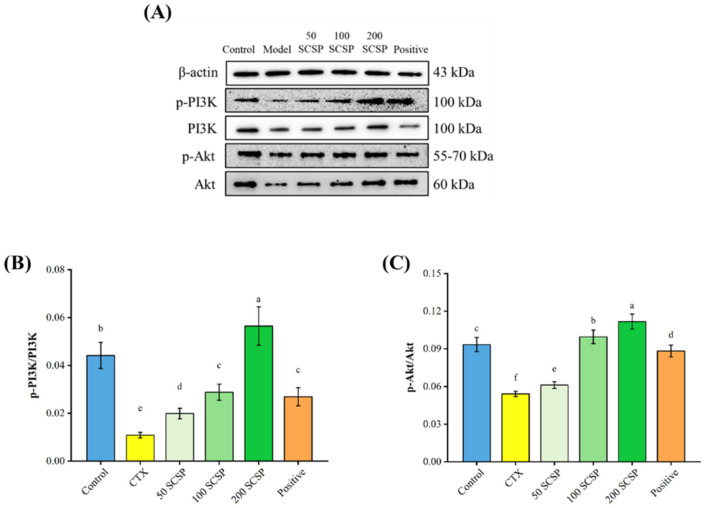
Effect of SCSP on the splenic PI3K/Akt pathway in mice (*n* = 10). (**A**) Western blotting of the related proteins in the PI3K/Akt pathway; (**B**) The expression of p-PI3K/PI3K; (**C**) The expression of p-Akt/Akt.

**Figure 6:**
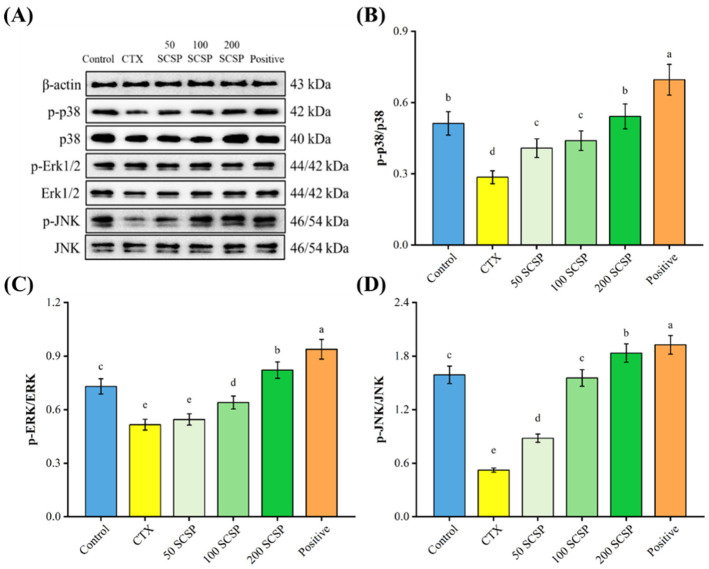
Effect of SCSP on the splenic MAPK pathway in mice (*n* = 10). (**A**) Western blotting of the related proteins in the MAPK pathway; (**B**) the expression of p-p38/p38; (**C**) the expression of p-ERk/ERk; (**D**) the expression of p-JNK/JNK.

To explore the mechanisms underlying the immunomodulatory effects of SCSP, the expression levels of proteins associated with the MAPK/NF-κB and PI3K/Akt pathways were analyzed in the spleen. In both pathways, CTX significantly inhibited the phosphorylation levels of the corresponding proteins (*p* < 0.05). After the administration of 200 mg/kg SCSP, the proportions of p-PI3K/PI3K and p-Akt/Akt were dramatically up-regulated in comparison with the CTX group (Figure 5B,C, *p* < 0.05), and the levels of up-regulation were higher than in the positive control group. Moreover, a notable enhancement in the phosphorylation levels of JNK, ERK and p38 was detected when SCSP (200 mg/kg) was administered (Figure 6B–D, *p* < 0.05).

**Figure 7:**
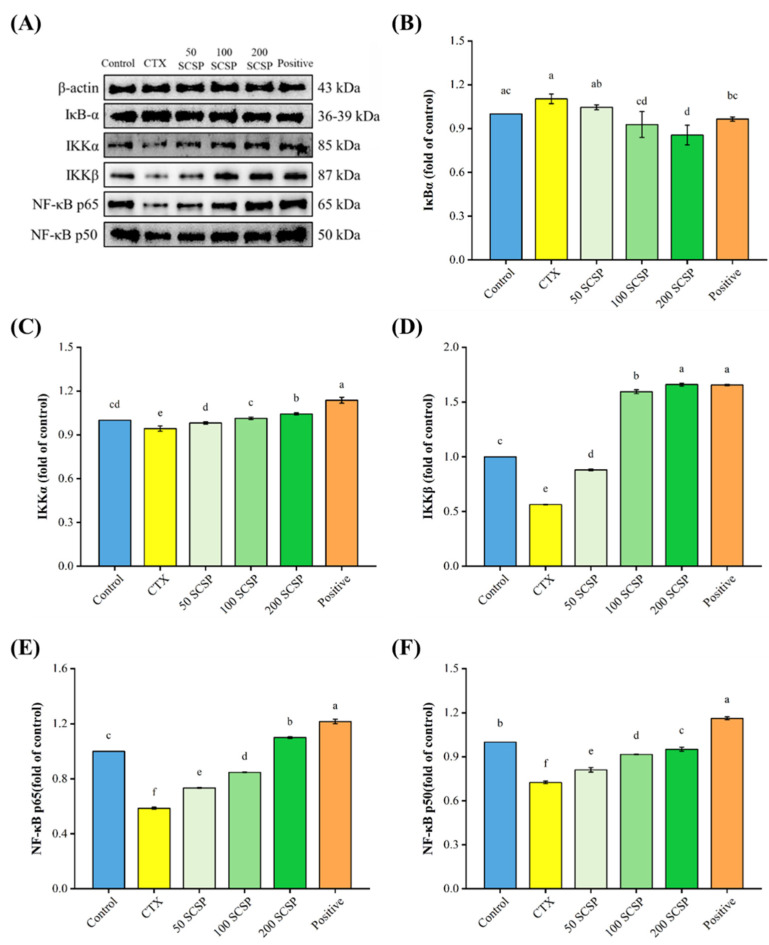
Effect of SCSP on the splenic NF-κB pathway in mice (*n* = 10). (**A**) Western blotting of the related proteins in the NF-κB pathway; (**B**) the expression of IκBα; (**C**) the expression of IKKα; (**D**) the expression of IKKβ; (**E**) the expression of NF-κB p65; (**F**) the expression of NF-κB p50.

As shown in Figure 7, NF-κB p50, NF-κB p65, IKKα, and IKKβ protein expression levels were markedly down-regulated after CTX treatment, while the expression level of IκBα increased, but not significantly. After treatment with 200 mg/kg of SCSP, the protein levels of NF-κB p50, NF-κB p65, IKKα, and IKKβ in the spleen of immunosuppressed mice increased considerably. Meanwhile, the expression of IκBα was clearly down-regulated (*p* < 0.05) when compared with the control. These results suggest that SCSP exerts its immunomodulatory effects in mice by activating the MAPK/NF-κB and PI3K/Akt pathways.

### 2.5. Expression of NF-κB p65 in the Spleen

**Figure 8:**
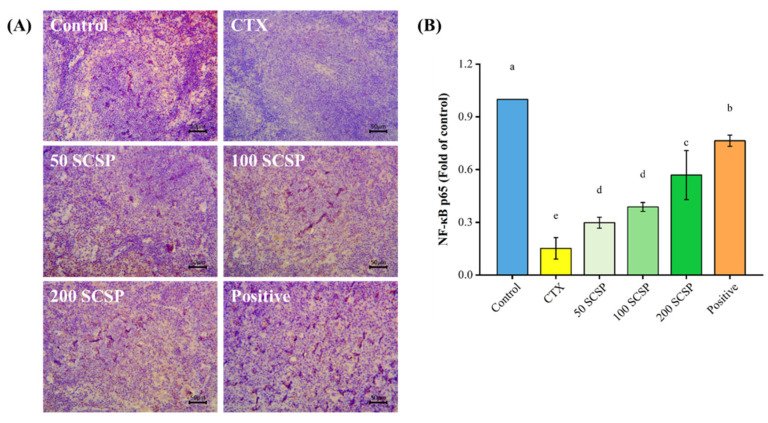
Effect of SCSP on NF-κB p65 expression in the spleen of mice (*n* = 10). (**A**) Immunohistochemistry of the spleen (×200); (**B**) semi-quantitative analysis of NF-κB p65.

Immunohistochemical results showed that NF-κB p65 was highly expressed in the spleen of untreated mice, as evidenced by the brownish-yellow color in the cytoplasm and nucleus, whereas it was hardly observed in the CTX-immunosuppressed group. In contrast, the expression of NF-κB p65 increased gradually with higher SCSP doses (Figure 8). On the other hand, the expression level of NF-κB p65 in the positive control group was between that of the control and the SCSP-treated groups. These findings indicate that SCSP can increase NF-κB p65 expression in the spleen of CTX-immunosuppressed mice.

### 2.6. Expression of p-PI3K in Spleen

**Figure 9:**
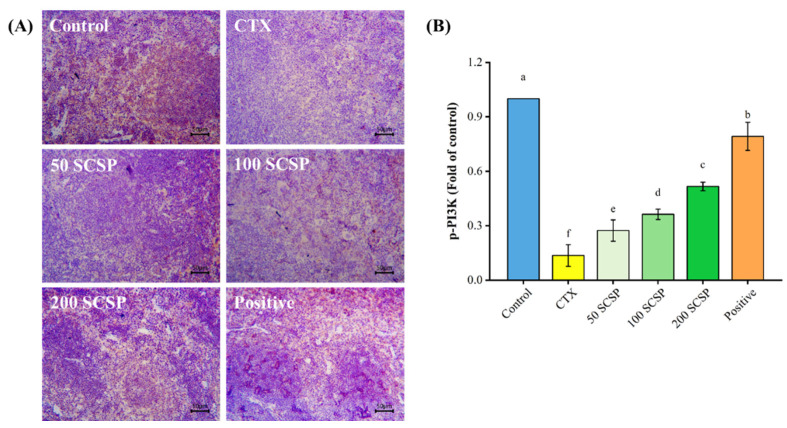
Effect of SCSP on p-PI3K expression in the spleen of mice (*n* = 10). (**A**) Immunohistochemistry of the spleen (×200); (**B**) semi-quantitative analysis of p-PI3K.

The impact of SCSP on the expression of p-PI3K in the spleen of immunosuppressed mice is shown in Figure 9. The expression of p-PI3K in immunosuppressed mice was significantly lower than that in the untreated control group, as evidenced by the almost complete disappearance of brown granules in the cytoplasm. In contrast, some recovery of brown particles was observed following administration of SCSP (200 mg/kg), suggesting that SCSP can enhance the weak expression of p-PI3K caused by CTX treatment.

### 2.7. Expression of p-p38 in the Spleen

**Figure 10:**
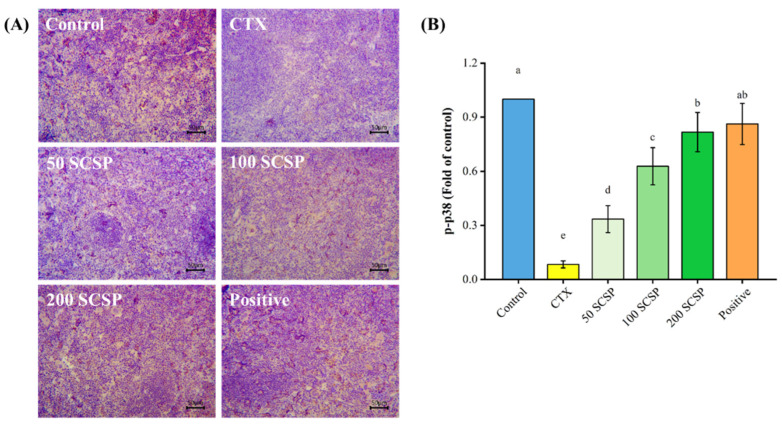
Effect of SCSP on p-p38 expression in the spleen of mice (*n* = 10). (**A**) Immunohistochemistry of the spleen (×200); (**B**) semi-quantitative analysis of p-p38.

The expression of p-p38 in the spleen was also analyzed, and this was seen as a brownish-yellow region. Unlike the control group, the p-p38 protein was minimally expressed in the spleen of immunosuppressed mice (Figure 10A). However, it can be seen in the image that the brown particles in the SCSP and positive control groups clearly increased, and there was no noticeable difference between these two groups.

## 3. Discussion

The immune system is an intricate network of immune organs, cells, and active substances that interact with each other to maintain the healthy operation of the body [1]. The rigorous regulation of the immune system is essential to ensure that the body mounts an appropriate response to pathogens while preventing excessive immune reactions. With the discovery of immunomodulators, immune regulation as a therapeutic approach against tumors, autoimmune diseases, and inflammatory diseases has become a reality [30]. CTX is the most commonly used chemotherapeutic drug against cancer in clinical practice, but it is associated with unwanted side effects such as immunosuppression [31]. To characterize an immunomodulator that can counteract this unwanted effect, we established a murine model of immunosuppression by intraperitoneally administering 80 mg/kg CTX for three continuous days. We used this model to explore the potential immunomodulatory mechanisms of SCSP.

Consistent with previous results [20], the suppression of immune function induced by CTX was reflected in the body weight and immune organ indices. The production of serum IL-1β, IL-6 and TNF-α, and the expression of NF-κB p65, p-PI3K and p-p38 in the spleen were all considerably lower than in the control group. Moreover, under the influence of CTX, the proliferative and phagocytic abilities of peritoneal macrophages and the proliferation of spleen lymphocytes were reduced, indicating that the CTX-induced immunosuppression model was successful.

As the largest immune organ in the body, the spleen is not only part of the lymphatic system, but also an important site for lymphocytes to migrate and receive antigenic stimulation to generate immune responses and immune effector molecules [32]. The thymus is the site for the differentiation, development and maturation of T lymphocytes. The development of all lymphoid organs and the generation of immunity in the body require the replenishment of T lymphocytes [28]. CTX can trigger immune organ atrophy and weight loss by reducing lymphocyte numbers in immune organs and inhibiting their proliferation and differentiation [31,33]. Our results showed that SCSP alleviated weight loss, splenic and thymic atrophy, and increased immune organ indices in immunosuppressed mice, suggesting that SCSP treatment has a significant immune-enhancing effect on immune organs.

Cytokines are small-molecular-weight proteins synthesized and secreted by immune cells. They perform critical functions, regulating cell interactions and the growth and differentiation of immune cells during immune responses [34]. It has been reported that polypeptides can stimulate several cellular immunological responses and regulate the secretion of different cytokines, thereby enhancing immune function in mice [35,36,37]. Our findings demonstrated that serum cytokines (IL-1β, IL-6, and TNF-α) in SCSP-treated mice increased in a dose-dependent manner. The lymphocyte is a major player in the immune response. T cells and B cells mediate cellular and humoral immunity, respectively, and their proliferation is directly correlated with the strength of specific immune responses [38]. Macrophages are involved in the recognition, phagocytosis and degradation of pathogens and trigger adaptive immune responses by presenting antigens to T cells [39]. Furthermore, in the initial stages of inflammation, macrophages play an indispensable role by releasing cytokines and chemokines [40]. Compared with the CTX-immunosuppressed group, SCSP significantly restored the proliferative rate of lymphocytes (T and B cells) and macrophages. Moreover, with increasing SCSP doses, the phagocytic capacity of peritoneal macrophages also increased. These results suggest that SCSP treatment reverses CTX-induced immune damage by enhancing immune cell function in mice.

NF-κB is a family of transcription factors involved in various biological processes such as inflammation, apoptosis, and proliferation [41,42]. The typical NF-κB pathway is considered to be a central regulator of inflammatory responses and has been extensively studied in human autoimmune diseases and cancer [43]. Akt serves as a key component of the PI3K/Akt signaling pathway, mediating multiple cellular functions, including metabolism, growth, and proliferation [44]. The activation of upstream PI3K enables Akt to regulate NF-κB signaling by phosphorylating IKK [45]. Our results showed that SCSP not only up-regulated the expression of important components of the NF-κB pathway in the spleen (including IKKα, IKKβ, NF-κB p50 and p65), but also enhanced the phosphorylation levels of PI3K and Akt proteins, and these results were consistent with the immunohistochemical results in splenic tissue. As one of the important pathways in the eukaryotic signal transmission network, the MAPK pathway modulates a number of crucial cellular physiological processes such as cell proliferation, differentiation, and inflammatory responses [46]. Western blotting results indicated that SCSP treatment of CTX-immunosuppressed animals increased the phosphorylation of JNK, ERK, and p38 proteins when compared with the CTX-immunosuppressed group. In addition, the immunohistochemical results also indicated that the SCSP group showed high expression of p-p38 protein. It has previously been reported that wild-simulated ginseng can activate mouse macrophages to produce immunomodulators (TNF-α, IL-1β, and IL-6) and intensify phagocytosis via the MAPK, NF-κB, and PI3K/Akt pathways [47]. Our overall results are consistent with the above reports, since the immune-enhancing effects of SCSP in CTX-immunosuppressed mice was achieved through activation of the MAPK/NF-κB and PI3K/Akt pathways.

## 4. Materials and Methods

### 4.1. Materials and Reagents

SCSP was provided by Wuxi MimoTopes Biotechnology (Wuxi, China) [20]. CTX was purchased from Hengrui Medicine (Lianyungang, China). The DAB immunohistochemistry kit was purchased from Boster (Wuhan, China). Neutral red staining solution and primary antibodies against β-actin, NF-κB p50, NF-κB p65, IKKα, IKKβ, and IκBα were supplied by Beyotime (Shanghai, China). The remaining primary antibodies were provided by Cell Signaling Technology Inc. (Beverly, MA, USA).

### 4.2. Animals and Treatment

**Figure 11:**
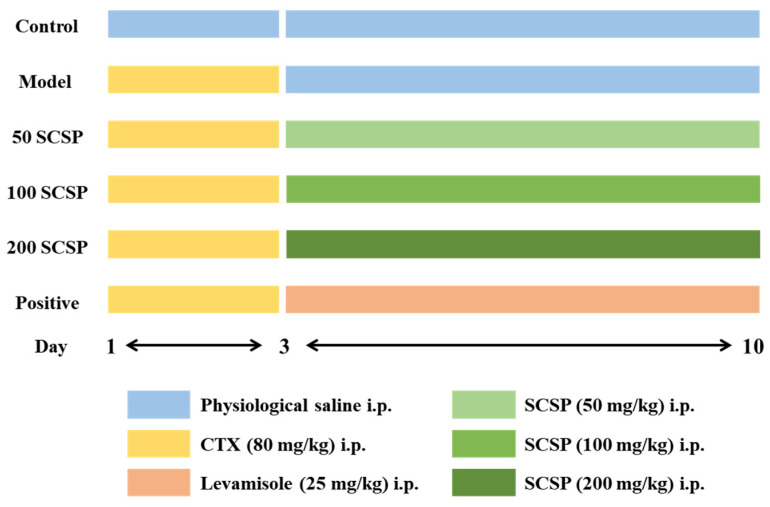
The experimental scheme and treatment of mice (*n* = 10). i.p., intraperitoneal injection.

A total of 60 male ICR mice (six-week-old, 20 ± 2 g) were purchased from the Zhejiang Academy of Medical Sciences (Hangzhou, China). All procedures in laboratory animals were authorized by the Animal Ethics Committee of Zhejiang Ocean University (SCXK ZHE 2019-0031). Mice were randomly assigned to six groups (*n* = 10) after the one-week adaptation period. With the exception of the control group, the remaining groups were treated with 80 mg/kg CTX continuously for three days [48]. Subsequently, the experimental groups were treated with different doses of SCSP (50, 100, and 200 mg/kg), while the positive control group received levamisole hydrochloride (25 mg/kg) at the same time for seven consecutive days (Figure 11). Twenty-four hours after the last feeding, blood samples were obtained using eyeball extirpation, and the mice were sacrificed by cervical dislocation.

### 4.3. Immune Organ Indices

The daily weight fluctuations of mice were monitored and recorded during the whole experiment. The collected spleens and thymuses were used to determine the organ indices using the following formula:

Thymus or spleen index = thymus or spleen weight (mg)/body weight (g)(1)

### 4.4. Cytokines Assays in Serum

Blood samples were collected in centrifuge tubes without anticoagulant treatment and placed in a refrigerator at 4 °C. After blood coagulation, serum was collected by centrifuging (6000× *g*, 5 min). The concentrations of IL-6, IL-1β, and TNF-α were determined following the guidelines by Solarbio (Beijing, China).

### 4.5. Splenic Lymphocyte Proliferation Assay

To evaluate the proliferative responses of T and B lymphocytes, splenic lymphocytes were stimulated with Con A and LPS, respectively [49,50]. The preparation of mouse spleen lymphocytes was carried out as described by Tang et al. [49,50]. The collected cells were seeded in a 96-well plate (1 × 10^6^ cells/mL, 4 replicate wells in each group), Con A (5 μg/mL) or LPS (1 μg/mL) was added, and the plate was placed in an incubator (Forma 3111 CO_2_ incubator, Thermo Forma, Waltham, MA, USA) at 37 °C with 5% CO_2_ for 24 h. Then, 200 μL of MTT staining solution was added, and 150 μL of DMSO was added after incubating for 4 h. The absorbance at 570 nm was measured (SpectraMax M2 microplate reader, Molecular Devices, Silicon Valley, CA, USA).

### 4.6. Peritoneal Macrophage Proliferation Assay

The mice were intraperitoneally injected with sterile saline solution (5 mL), and the abdomen was gently pressed for 2 min, and the abdominal wall was cut open. Then, the abdominal fluid was sucked into a centrifuge tube, and the cell suspension was centrifuged (2000× *g*, 10 min) and resuspended with DMEM medium. After incubation at 37 °C with 5% CO_2_ for 4 h, the supernatant was discarded to obtain purified macrophages [49,50]. The cell density was then adjusted (1 × 10^4^ cells/mL), seeded in 96-well plates (200 μL per well), and incubated for 24 h. After discarding the supernatant from each well, 200 μL of PBS containing 10% MTT was added, and the plates were incubated for another 4 h. Then, 150 μL DMSO was added, and the optical density (OD) at 570 nm was measured to calculate the proliferation rate of macrophages.

### 4.7. Macrophage Phagocytic Capacity

Macrophage phagocytosis was examined by measuring neutral red uptake [50]. The collected peritoneal macrophages were seeded on a 96-well plate (5 × 10^5^ cells/mL) and incubated for 24 h. Then, 200 μL of nutrient solution (excluding NaHCO_3_), and 20 μL of neutral red staining solution was added. The supernatant was discarded after incubating for 2 h, lysis buffer was added, and the cells were incubated for another 10 min. The absorbance at 540 nm was measured, and the phagocytic index was calculated.

### 4.8. Western Blotting

The experimental procedures were conducted as previously described [31]. Briefly, the BCA protein assay kit was used to measure the amount of protein in each spleen homogenate supernatant, and 30 μg of proteins were loaded per lane. The proteins were separated using a 12% SDS-PAGE gel and then transferred to the PVDF membrane. Enhanced chemiluminescence (ECL) was utilized to detect the bands, and data processing was carried out using the Alphaview SA gel image analysis software (Fluor Chem FC3, ProteinSimple, San Jose, CA, USA).

### 4.9. Immunohistochemical Analysis

Paraffin sections of the mouse spleen were deparaffinized and rehydrated. Then, endogenous peroxidase was blocked with H_2_O_2_ and treated with antigen retrieval solution. After incubating with the primary antibody at 4 °C overnight, the secondary antibody was added, and the samples were incubated for 1 h. Finally, the DAB immunohistochemical staining kit was used for color development, and the staining characteristics of each group were analyzed with a CX31 biological microscope (Olympus, Tokyo, Japan).

### 4.10. Statistical Analysis

One-way analysis of variance (ANOVA) was performed on the experimental data using SPSS 22.0 software. All results are expressed as the mean ± standard deviation (x ± SD) and differences between means were considered significant at *p* < 0.05.

## 5. Conclusions

**Figure 12:**
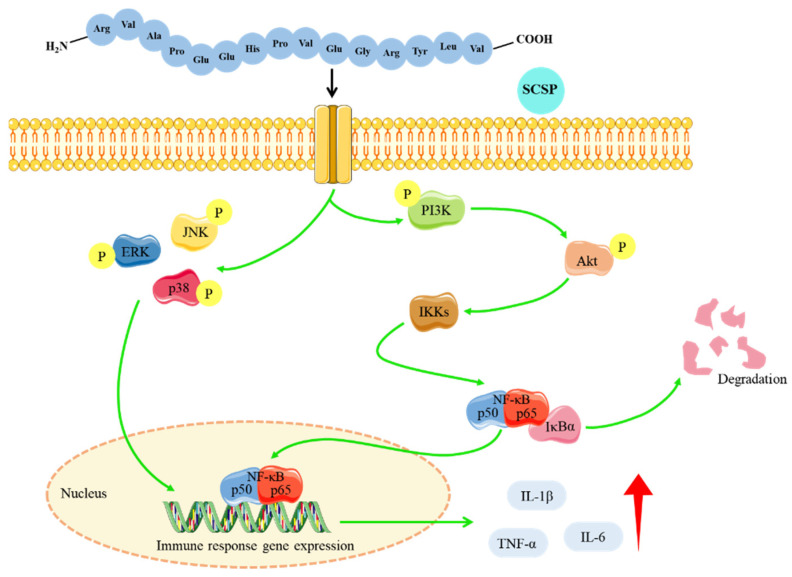
SCSP ameliorates CTX-induced immunosuppression possibly by regulating the MAPK/NF-κB and PI3K/Akt pathways.

In conclusion, SCSP enhanced immune responses by attenuating CTX-induced splenic and thymic damage in mice, enhancing the cellular functions of splenic lymphocytes and peritoneal macrophages, and promoting cytokine secretion. Moreover, SCSP activated the MAPK/NF-κB and PI3K/Akt pathways to enhance murine immunity (Figure 12). Our findings suggest that SCSP can effectively reverse CTX-induced murine immunosuppression, indicating that SCSP could be developed as a new immunomodulator or immune adjuvant in the future.

## Data Availability

Data supporting our findings can be sent upon request.
